# Compensatory Neural Reorganization in Tourette Syndrome

**DOI:** 10.1016/j.cub.2011.02.047

**Published:** 2011-04-12

**Authors:** Stephen R. Jackson, Amy Parkinson, Jeyoung Jung, Suzanne E. Ryan, Paul S. Morgan, Chris Hollis, Georgina M. Jackson

**Affiliations:** 1 WCU Department of Brain and Cognitive Engineering, Korea University, Seoul 136-713, South Korea; 2 School of Psychology, The University of Nottingham, Nottingham NG7 2RD, UK; 3 Division of Psychiatry, The University of Nottingham, A Floor, South Block, Queen's Medical Centre, Nottingham NG7 2UH, UK; 4 Department of Academic Radiology, The University of Nottingham, Nottingham NG7 2UH, UK

## Abstract

Children with neurological disorders may follow unique developmental trajectories whereby they undergo compensatory neuroplastic changes in brain structure and function that help them gain control over their symptoms [1, 2, 3, 4, 5, 6]. We used behavioral and brain imaging techniques to investigate this conjecture in children with Tourette syndrome (TS). Using a behavioral task that induces high levels of intermanual conflict, we show that individuals with TS exhibit enhanced control of motor output. Then, using structural (diffusion-weighted imaging) brain imaging techniques, we demonstrate widespread differences in the white matter (WM) microstructure of the TS brain that include alterations in the corpus callosum and forceps minor (FM) WM that significantly predict tic severity in TS. Most importantly, we show that task performance for the TS group (but not for controls) is strongly predicted by the WM microstructure of the FM pathways that lead to the prefrontal cortex and by the functional magnetic resonance imaging blood oxygen level-dependent response in prefrontal areas connected by these tracts. These results provide evidence for compensatory brain reorganization that may underlie the increased self-regulation mechanisms that have been hypothesized to bring about the control of tics during adolescence.

## Highlights

► We combine behavioral studies with structural and functional neuroimaging ► We report enhanced cognitive control in children with Tourette syndrome ► Frontal lobe white-matter microstructure predicts tic severity in Tourette syndrome ► Frontal lobe white-matter and fMRI BOLD predict cognitive control in Tourette syndrome

## Results

Tourette syndrome (TS) is a developmental neurological disorder characterized by the presence of chronic vocal and motor tics [7]. Tics are involuntary, repetitive, stereotyped behaviors that occur with a limited duration, typically many times in a single day.

The neurological basis of TS is unclear at this time; however, it is generally agreed that the basal ganglia, including circuits that link the striatum to cortex, are dysfunctional [8]. However, structural or functional changes observed within the TS brain can reflect causes and/or consequences of the TS disorder, and it has been proposed that cortical reorganization of behavioral control circuits may operate to compensate for TS [1, 2, 3, 4, 5, 6]. Thus, it is suggested that children and adolescents with TS gain control over their tics through the development of compensatory self-regulation mechanisms, most likely implemented within neural circuits linking prefrontal cortex with primary and secondary motor regions [1, 2, 3, 4, 5, 6]. Behavioral evidence supporting this suggestion comes from the finding that individuals with “pure” TS (i.e., without comorbid attention deficit hyperactivity disorder [ADHD]) exhibit paradoxically enhanced levels of cognitive control over their oculomotor responses compared to a group of age-matched typically developing individuals [4, 5] and that this enhanced oculomotor performance is highly associated with clinical measures of tic severity [5].

### Experiment 1: Behavioral Study

**Figure 1:**
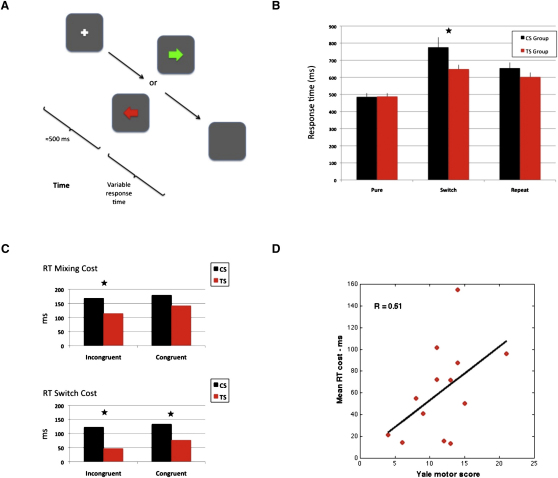
Intermanual Conflict Task and Results (A) Graphic representation of the behavioral task-switching paradigm. After fixating a white cross, participants were presented with a colored arrow. If the arrow was green, they executed a manual response with the hand indicated by the direction of the arrow. If the arrow was red, they executed a manual response with the opposite hand. (B) Mean response times (ms) for the Tourette syndrome (TS) and control (CS) groups for manual responses executed within “pure” and “mixed” blocks. Error bars are standard errors. (C) Response time (RT) “mixing” and “switch” costs (see text for details) for the TS and CS groups. (D) Scatter plot illustrating the relationship between tic severity (as measured by Yale motor score) and mean RT costs. Despite the relatively small number of individuals, the analysis revealed a strong positive correlation (R = 0.51, p = 0.07).

To examine behavioral regulation of motor output in children and adolescents with TS, we used a manual task-switching paradigm that induced high levels of interhemispheric conflict (Figure 1A). This task was administered to a group of individuals with TS (but without comorbid ADHD) and a group of age- and gender-matched typically developing children. Response times (RT) and error rates were obtained for separate blocks of “pure” and “mixed” blocks (Figure 1B).

A priori, between-group contrasts revealed that performance in the pure blocks was equivalent across groups. By contrast, analysis of the “mixed” trials confirmed that RT for the TS group was significantly faster than that of controls on switch trials (t = 2.1, p < 0.05), but not for repeat trials (t = 1.2, p > 0.1). Error rates did not differ between groups for mixed trials (p > 0.1). This finding is consistent with previous studies of oculomotor task switching in TS [5, 6]. Between-group analyses also confirmed that mixing costs and switch costs were significantly reduced in the TS group (Figure 1C).

Finally, additional analyses revealed that tic severity (Yale motor score [9]) was highly positively associated with RT switch costs (Figure 1D). Thus, individuals with increased cognitive control and low switch costs exhibit reduced levels of tic severity. These data support the hypothesis that children with TS exhibit enhanced self-regulation of motor outputs compared to typically developing children.

### Experiment 2: Diffusion-Weighted Imaging Study

The above hypothesis predicts that increased levels of cognitive control may be accompanied by white matter (WM) changes in individuals with TS. WM microstructure was investigated via diffusion-weighted imaging (DWI), which is sensitive to diffusion of water in brain tissue. Two measures were obtained at each voxel: fractional anisotropy (FA) and mean diffusivity (MD). These measures index key properties of WM including myelination and density of axonal fibers [10, 11]. DWI scans were acquired from 14 children with TS and 14 age- and gender-matched typically developing children (see Supplemental Information available online).

**Figure 2:**
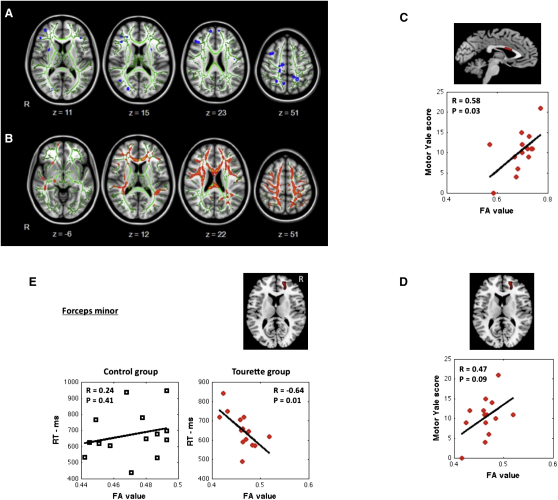
Diffusion-Weighted Imaging Results (A) Group (n = 28; 14 TS and 14 CS) differences in fractional anisotropy (FA) values. Whole-brain analyses using tract-based spatial statistics demonstrated widespread statistically significant decreases in FA in the TS group that included the corticospinal tract and the long association fibers. Group differences are superimposed onto the Montreal Neurological Institute 152 brain. The mean tract skeleton is displayed in green, and all significant (p < 0.01) decreases in FA are displayed in blue. (B) Group differences in mean diffusivity (MD). The images show widespread significant increases in MD in the TS group compared to the controls (p < 0.01) displayed in red on the MNI 152 brain and mean tract skeleton (green). (C) Scatter plot illustrating the strong linear relationship in the TS group between tic severity (Yale) and the microstructure (FA) within the mid-corpus callosum WM tract. (D) Scatter plot illustrating the strong linear relationship in the TS group between tic severity (Yale) and the microstructure (FA) within the right forceps minor WM tract. (E) Scatter plot for each group illustrating the linear relationship between FA values within the right forceps minor WM tract and RT performance on the manual task-switching paradigm (see text for details).

Whole-brain comparison of FA and MD demonstrated that there were widespread differences in the TS group (Figures 2A and 2B) compared to controls (p < 0.01, family-wise error corrected). The TS group exhibited reduced FA values and/or increased MD, particularly in the corpus callosum (CC) and forceps minor (FM, a WM pathway that connects the lateral and medial areas of the prefrontal cortex). There were no regions of significantly increased FA or decreased MD for the TS group. These results are consistent with previous reports of altered WM in individuals with TS, particularly in the corpus callosum and frontal WM pathways [1, 2, 6, 12, 13, 14, 15, 16, 17].

#### Correlation with Clinical Scores

Linear associative analyses revealed that the WM microstructure (FA and MD) in the CC and the right FM was strongly associated with clinical measures (Yale motor score) of tic severity (Figures 2C and 2D). FA values showed a strong positive association with tic severity (R ≥ 0.47). Additional analyses confirmed that this association was independent of age (see Supplemental Information).

Changes in WM might be a core component of the TS disorder or could reflect the results of a compensatory functional reorganization in response to the disorder [1, 2]. Importantly, whereas the TS group exhibit reduced FA values compared to controls, relative increases in FA within the TS group were themselves associated with increased tic severity scores.

A study examining interhemispheric connectivity in TS previously reported that the size of the CC was significantly reduced in children with TS and that CC size was inversely related with the volumes of frontal cortex [2]. This study also reported that CC size was positively correlated with clinical measures of tic severity, indicating that relative increases in CC size are associated with greater tic severity and not less [2]. This result, together with our own finding, indicates that changes in cortical WM most likely reflect a functional adaptation to TS rather than a core symptom of the disorder. That said, we acknowledge that the observed WM differences could alternatively reflect individual variation (genetic or ontogenetic) in connectivity that might confer on some individuals with TS an increased capacity to suppress tics. A longitudinal study would distinguish between these two possibilities.

#### Correlation with Behavioral Task Performance

We examined whether performance on the behavioral task was predicted by WM in the CC. This was the case for the control (CS) group (Pearson correlation between CC MD values and incongruent switch RT: R = 0.59, p < 0.03; incongruent switch costs: R = 0.62, p < 0.02), but not for the TS group. For the CS group only, long RTs and high switch costs were positively associated with higher diffusivity in the CC and negatively associated with CC FA. Additional analyses confirmed that this association between WM microstructure and RT performance was independent of age (see Supplemental Information).

By contrast, whereas RT performance in the TS group was not strongly associated with the WM of the CC (incongruent switch RT: R = 0.23, p = 0.4; incongruent switch costs: R = 0.13, p < 0.67), it was predicted by the WM microstructure of the FM (but only for the TS group; see Figure 2E). Statistical testing of the difference between correlation coefficients for the CS group (0.24) and TS group (−0.64) revealed that this difference was statistically significant (Z = 2.35, p < 0.05). Additional analyses confirmed that the association between WM microstructure and RT measures was independent of age (see Supplemental Information). These results provide important support for the hypothesis that for the TS group, the normal role performed by the CC in resolving the selection of motor outputs may be reduced in favor of an increased role for prefrontal cortex.

### Experiment 3: Functional Magnetic Resonance Imaging Study

We used functional magnetic resonance imaging (fMRI) to investigate differences in the functional anatomy of the TS brain while participants performed mixed blocks of the manual task-switching paradigm outlined in experiment 1. Ten young individuals with TS but without comorbid ADHD participated in the study. The control group compromised 15 age-matched typically developing children (see Supplemental Information for additional details). Analyses of the RT responses confirmed that both groups exhibited typical task-switching effects—RTs were substantially slower for incongruent compared to congruent trials (p < 0.005) and slower for task switch compared to task repeat trials (p < 0.001). There were no statistically significant differences in RT between the groups for any of the experimental conditions (p > 0.1).

**Figure 3:**
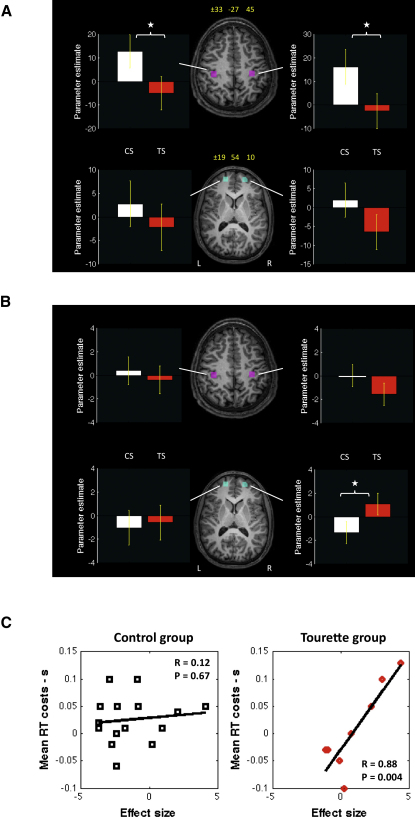
fMRI Results (A) fMRI blood oxygen level-dependent (BOLD) response for the all conditions > rest contrast within each bilateral 10 mm^3^ region of interest (ROI), defined on anatomical and functional grounds, within the hand area of primary motor (purple, top panel) and prefrontal (cyan, bottom panel) cortex (Talairach coordinates for ROI centroid are shown in yellow). White bars indicate average BOLD activation for the CS group; red bars indicate average BOLD activation for the TS group. Error bars are standard errors. (B) fMRI BOLD response associated with the incongruent switch condition for ROIs within the hand area of primary motor (purple, top panel) and prefrontal (cyan, bottom panel) cortex. White bars indicate the average BOLD activation for the CS group; red bars indicate average BOLD activation for the TS group. Error bars are standard errors. (C) Scatter plot for each group illustrating the linear relationship between fMRI BOLD responses within the right prefrontal cortex ROI and individual RT cost values for the incongruent condition of the manual task-switching paradigm.

Given the results of the DWI study reported above, the analyses of the fMRI blood oxygen level-dependent (BOLD) data reported here were directed solely toward determining whether there were significant differences in the BOLD response for the TS group, relative to a group of age-matched controls, in regions of cortex associated with (A) the mid CC or (B) the vicinity of the FM. Regions of interest (ROIs, 10 mm^3^) were determined for the hand area of the primary motor cortex in each hemisphere and for an area of prefrontal cortex immediately adjacent to the right FM (see Supplemental Information). The locations of these ROIs are displayed on the spatially normalized anatomical MRI of a single participant in Figure 3A.

To examine overall between-group differences in BOLD activation in the motor and prefrontal ROIs, we conducted a random-effects (RFX) analysis for the all conditions > rest contrast (Figure 3A). To examine between-group differences in the BOLD activation associated with cognitive control, we followed the procedure adopted in a recent fMRI study of task-set switching in TS children [18] and examined the BOLD activation for the most difficult behavioral condition—the incongruent-switch condition (Figure 3B).

#### All Conditions > Rest

The analyses revealed that for the all conditions > rest contrast, the fMRI BOLD response in the left and right hemisphere motor cortex ROIs was significantly greater for the CS group (left M1 ROI: t = 2.53, p < 0.02; right M1 ROI: t = 2.56, p < 0.02). By contrast, the mean BOLD response in the prefrontal cortex ROIs for this contrast did not differ between the groups. Means for this contrast are presented in Figure 3A.

#### BOLD Response: Incongruent-Switch Trials

##### Motor Cortex ROIs

The RFX analyses confirmed that the BOLD response in the motor cortex ROI was significantly larger for congruent compared to incongruent trials (−36.0 ± 12 arbitrary units [AU], t = −2.96, p < 0.003) and for task repeat compared to task switch trials (−22.3 ± 12 AU, t = −1.83, p = 0.07). This finding is consistent with previous event-related potential studies of task switching [19] and suggests that the motor cortex BOLD response largely reflects ease of motor execution, or motor fluency, and is reduced in task conflict situations. Importantly, the between-group RFX analyses revealed that for the motor cortex ROIs, the BOLD response on incongruent switch trials was not significantly different for the CS and TS groups (means: CS group = 0.21 ± 1.1 AU, TS group = −0.98 ± 1.1 AU; t = 1.1, p = 0.28), suggesting that motor execution was equivalent across the groups.

##### Prefrontal ROIs

The RFX analyses confirmed that, in contrast to the motor cortex ROIs, the BOLD response in the prefrontal cortex ROIs was significantly larger for incongruent compared to congruent trials (68.4 ± 12.2 AU, t = 5.6, p < 0.00001), indicating that, as might be expected from previous studies, the fMRI BOLD response in the prefrontal cortex increases in task conflict situations [20]. Further analyses revealed that for the right hemisphere inferior frontal ROI, the between-group contrast for incongruent-switch trials was statistically significant for the right hemisphere prefrontal ROI, which exhibited an increased BOLD response in the TS group during the high-conflict incongruent-switch trials relative to incongruent-repeat trials (t = −2.63, p < 0.02). Relevant means are presented in Figure 3B. This finding is consistent with converging evidence from electrophysiological recording studies in the monkey and neuropsychological and brain imaging studies in humans that the right prefrontal cortex is implicated in inhibitory processes underlying task switching [20, 21].

#### Correlation between Prefrontal BOLD and Behavioral RT Costs

To investigate the relationship between the fMRI BOLD response in the right prefrontal cortex ROI and behavior in the manual task-switching paradigm, we examined the linear relationship, for each group, between individual RT cost values (switch minus repeat trials) for the incongruent condition and individual parameter estimates for the appropriate fMRI BOLD contrast (i.e., incongruent switch trials > incongruent repeat trials). These data are presented in Figure 3C. This figure illustrates that for the TS group only, the fMRI BOLD response was very strongly and positively linearly associated with RT costs (R = 0.88, p = 0.004). Thus, increased BOLD activation in the right prefrontal ROI is associated with high-conflict situations and, in the TS group, with increased RT costs. In contrast, the linear relationship between the BOLD response in the right prefrontal cortex ROI and RT switch costs was largely absent for the control group and was not statistically significant (R = 0.12, p = 0.67). Additional analyses confirmed that a similar pattern of results was observed for the TS and CS groups in the left hemisphere prefrontal cortex ROI (see Supplemental Information). These data confirm that for the TS group only, the prefrontal cortex is significantly involved in the cognitive control of motor outputs.

## Discussion

The premise explored in this paper is that children with neurological disorders can follow unique developmental trajectories and undergo compensatory changes in brain structure and function, particularly within prefrontal cortex [1, 2, 3] and corpus callosum [1, 2, 6], that allow them to gain control over their symptoms. We used functional and structural brain imaging techniques to provide strong support for this suggestion.

First, we demonstrate that a group of children with “pure” TS exhibit enhanced control over their manual responses on a task-switching paradigm that induces high levels of intermanual conflict. This finding supports the proposal that a constant requirement to suppress tics may lead to a generalized increase in cognitive control over motor activity, including manual and saccadic responses [4, 5]. Importantly, we show that individual performance on the manual task-switching paradigm is strongly positively associated with tic severity.

Second, we report widespread alterations in WM microstructure for the TS group (characterized by reduced FA and increased MD relative to controls) and show that WM microstructure in the CC and the FM is strongly linearly associated with clinical measures of tic severity. This finding is consistent with previous reports of reduced FA and WM area in the CC [2, 6, 13, 16] and increased prefrontal cortex volume [1, 2, 6, 12] in children with TS. These findings are interpreted as evidence for neuroplastic adaptation in TS because CC area is inversely correlated with prefrontal cortex volume and also positively correlated with tic severity.

Importantly, we demonstrate that enhanced control of motor output in the TS group is predicted by the WM within the prefrontal cortex. The direction of the linear relationship between WM microstructure and tic severity suggests that these changes may be due to neuroplastic functional adaptation rather than a core component of the TS disorder. Thus, task performance in the control group, but not the Tourette group, is predicted by the WM microstructure of the CC, whereas task performance of the Tourette group, but not the control group, is significantly predicted by the WM microstructure of the FM.

Finally, using fMRI, we show that the BOLD response in the right prefrontal cortex immediately adjacent to the FM is significantly greater in the TS group compared to controls when carrying out the manual task-switching paradigm. Moreover, the fMRI BOLD response in the prefrontal cortex is strongly linearly related to behavioral (RT) performance on the manual task-switching paradigm for the Tourette group, but not for controls.

Taken together, these data provide strong evidence in support of the hypothesis that enhanced cognitive control of motor output in TS is accompanied by adaptive functional and structural changes in the prefrontal cortex.

## Experimental Procedures

Full details of the participants, methods, and procedures used in each of the experiments are provided in the Supplemental Information.

## Supplemental Information

Document S1. Supplemental Experimental Procedures, Supplemental Results, Supplemental Discussion, One Table, and Four Figures
