# Supplementary material for: Compensatory Neural Reorganization in Tourette Syndrome
Source: Curr Biol. 2011 Apr 12;21(7):580–5. doi: 10.1016/j.cub.2011.02.047 (PMC3076629; doi:10.1016/j.cub.2011.02.047)
Supplement: Document S1. Supplemental Experimental Procedures, Supplemental Results, Supplemental Discussion, One Table, and Four Figures [file mmc1.pdf]

Current Biology, Volume *21*

Supplemental Information

Compensatory Neural Reorganization in Tourette Syndrome

Stephen R. Jackson, Amy Parkinson, Jeyoung Jung, Suzanne E. Ryan, Paul S. Morgan, Chris Hollis, and Georgina M. Jackson

Study 1: Behavioral Study

Supplemental Experimental Procedures

Participants

The experimental group consisted of thirteen children with Tourette syndrome (11 male, 2 female, average age 14.2 years [174.2 months]). The control group comprised thirteen neurologically normal gender- and age-matched individuals (average age 14 years, 4 months). Patients were recruited through the Tourette syndrome clinic at the Queens Medical Centre, Nottingham. Participants who had a clinical diagnosis of ADHD or OCD were excluded from the sample. Current tic severity for the TS patients was assessed on the day of testing using the Yale Global Tics Severity Scale [R1]. The IQ of all participants was obtained using the Wechsler Abbreviated Scale of Intelligence (WASI) vocabulary and matrix reasoning subscales. Clinical data from the patient group is summarized in Table S1. All experiments were approved by the Nottingham Healthcare Trust and informed written consent was obtained from all individuals prior to participation.

Table S1. Clinical Details of TS Group

Subject ID	Sex	Age (months)	IQ	Global Yale Score (YGTSS)	Motor Yale Score	Medication	Tics (Description)
TS1	M	180	111	21	11	Clonidine 75 mcg	Coughing, grunting, eye blinking, head jerks
TS2	F	155	95	6	6	Clonidine 75 mcg	Eye blinking, nose movements
TS3	M	171	76	26	12	Clonidine 50 mcg	Facial grimace, grunting, coprolalia, repeating words
TS4	M	172	95	30	12	Clonidine 50 mcg	Hand movements, high pitched noises, echolalia
TS5	M	175	104	29	10	none	Eye blinking, throat clearing
TS6	M	214	101	7	4	none	Eye blinking, nose movements, screeching noises, echolalia
TS7	F	171	106	57	23	none	Head jerks, eye blinking, coughing, throat clearing
TS8	M	130	135	32	14	Clonidine 50 mcg	Nose movements, arm gestures, throat clearing
TS9	M	130	101	21	11	none	Eye blinking, eye movements, leg movements
TS10	M	217	112	55	15	Risperidone 1.5 mg	Eye blinking, nose movements, mouth movements, grunting
TS11	M	190	107	82	21	Fluoxetine 20 mg, Melatonin 3 mg, Aripipazole 5 mg	Eye blinking, mouth movements, facial grimce, head jerks, shoulder shrugs, coughing, throat clearing, sniffing, grunting, palalalia
TS12	M	143	120	19	9	none	Eye gestures, mouth movements, facial movements, throat clearing, sniffing, occasionally repeating sentences
TS13	M	217	123	28	11	none	Head jerks, leg movements, squeaking, saying things with an accent

Materials

Stimuli were displayed on a 17in Elo Touchscreen monitor with a spatial resolution of 640 x 480 pixels at a frame rate of 60Hz, at a viewing distance of 46cm. Stimuli were generated using the MATLAB (The MathWorks) Cogent Graphics toolbox developed by John Romaya at the Wellcome Department of Imaging Neuroscience. These stimuli consisted of a black fixation cross (Arial font, size 30) and two arrows (40 pixels in length and 20 pixels in height), one green and one red, presented on a grey background. Participants made leftward or rightward responses by pressing a key on the keyboard: 'z' for left and '/' for right. The participants' index fingers were positioned on the appropriate keys at the start of the experimental session so that they did not have to look away from the screen to locate them. The study was carried out in a darkened room.

Procedure

Participants completed a block of pure congruent (see below) trials, followed by a block of pure incongruent (see below) trials. On each of these pure blocks they were given three practice trials to familiarize them with the procedure. They then completed a block of mixed congruent and incongruent trials, followed by a further block each of pure congruent and incongruent trials. The details of these experimental blocks are outlined below.

Pure Blocks

Congruent Trials: At the start of each trial the fixation cross was presented at the centre of the screen. After a variable delay based on a normal distribution (mean = 500ms, SD = 125ms) an arrow was displayed centrally. A green arrow was then presented centrally. On half of the trials the arrow pointed to the left, and on the other half to the right. For a leftward pointing arrow participants were asked to make a leftward key press, and for a rightward arrow a rightward key press. There then followed a delay of 1500ms and the arrow was then removed from the screen ready for the start of the next trial. Approximately half of the trials were response repeat trials in which the response required, i.e. left or right key press was the same as for the preceding trial, whereas the remaining trials were response switch trials in which the side of the key press required (regardless of whether it was a congruent or incongruent) changed from that of the previous trial. The order of the response switch and repeat trials was pseudorandomly determined in advance by the computer, and was varied across participants. There were 48 trials in total, completed in 2 blocks of 24 trials each.

Incongruent Trials: These were identical to the congruent trials except in terms of the arrow color, which was red instead of green, and the response required. For a leftward pointing arrow participants were asked to make a rightward key press, and for a rightward arrow, to make a leftward key press.

Mixed Block

At the start of each trial the fixation cross was presented at the centre of the screen. After a variable delay based on a normal distribution (mean = 500ms, SD = 125ms) an arrow was displayed centrally. On half of the trials the arrow was green, and on the other half red. For half of the trials the arrow was leftward pointing, whereas on the other half it pointed to the right. The color of the arrow indicated the manual response required by the participant; green for a key press congruent with stimulus location, i.e. left for a leftward arrow or right

for a rightward arrow. A red arrow in contrast indicated an incongruent key press, i.e. right for a leftward arrow and left for a rightward arrow. There then followed a delay of 1500ms and the arrow was then removed from the screen ready for the start of the next trial. There were 96 trials in total, split into six blocks of 16 trials with a rest break in between each.

The experimental trials were counterbalanced such that there were an equal number of congruent and incongruent manual responses and an equal number of left and right responses. Approximately 50% of the trials (the first trial must be removed) were task repeat trials, in which the type of key press required, i.e. a congruent or incongruent was the same as on the preceding trial. The remaining trials were task switch trials in which the type of key press required differed from that of the previous trial. Further to this, approximately 50% of the trials were response repeat trials in which the response required, i.e. left or right key press was the same as for the preceding trial, whereas the remaining trials were response switch trials in which the side of the key press required (regardless of whether it was a congruent or incongruent) changed from that of the previous trial. The order of key press and response switch and repeat trials was pseudorandomly determined in advance by the computer, and was varied across participants.

Supplemental Results

Analyses of the 'mixed' trials confirmed that RTs were significantly longer on Switch trials compared to Repeat trials ($F[1,24] = 40.5$, $p < 0.0001$).

Supplemental Discussion

We have previously demonstrated, using an oculomotor response-switching task, that individuals with pure TS (i.e., without co-morbid disorders) exhibit paradoxically *greater* levels of cognitive control over their oculomotor responses than their age-matched controls. Specifically, the TS group exhibit significantly fewer errors on task-switch trials than controls: and this is not due to any speed-accuracy trade-off as their mean response times on task-switch trials are equivalent to those of controls [R2,R3].

One notable difference between the results of the current study and those of our previous oculomotor studies is that previously the effect on performance (i.e. the TS group performs significantly better than controls) was observed for error rates and not response latencies; whereas in the current study the effect is observed for response times and not errors. It is likely that this difference is due to the nature of the errors observed for oculomotor movements and the ease with which corrected errors can be observed for oculomotor movements, but not for manual responses. In our previous oculomotor studies, errors were defined as trials in which subjects executed a saccade to the incorrect target location, or began a movement toward the incorrect target location even if this was later corrected. Furthermore, in these studies the majority of the errors observed were in fact movements that were initially in the incorrect direction but were then successfully corrected. By contrast, in the current study errors were defined as trials in which the subject executed an incorrect response (i.e., pressed a response key with the incorrect hand). Thus, while it may have been the case that there were trials where subjects began an incorrect response but corrected it; such trials would not have been observed as errors. Instead, they may be reflected as a slowed response time. Future studies might examine this issue by using electromyographic (EMG) recording to detect corrected responses.

Study 2: Diffusion Tensor Imaging Study

Supplemental Experimental Procedures

Participants

Fourteen children with Tourette syndrome and Fourteen age- and gender-matched (12 male, 2 female, average age 15 years 4 months) typically developing children. All participants had completed the manual task-switching paradigm described in Experiment 1. Individuals who had a clinical diagnosis of ADHD or OCD were excluded from the sample. Current tic severity for the patients was assessed on the day of testing using the Yale Global Tics Severity Scale [R1].

MR Imaging

Image Acquisition: DTI data was acquired on a 3T Phillips Achieva Scanner (Phillips, Best, The Netherlands) using an 8-channel SENSE head coil. Whole brain DTI measurements were acquired using a single shot, spin echo, EPI diffusion tensor imaging sequence that lasted 6 minutes 51 seconds in total for each participant. Diffusion MR images were obtained with the following parameters: 32 directions; TR = 9062ms; TE = 57ms; b value = 1000s/mm^2; 52 contiguous axial slices; slice thickness = 2mm; FOV = 224x224mm. A b=0 image with no diffusion gradients was also obtained.

Image Preprocessing: *Images* were processed off-line using the FSL (FMRIB software library, http://www.fmrib.ox.ac.uk/fsl) Diffusion Toolkit (FDT). Eddy-current correction was performed on the spatially normalized diffusion weighted images to correct for distortions for different gradient directions, and for simple head motion, using affine registration to a reference volume. Diffusion tensors and maps of fractional anisotropy [FA], mean diffusivity [MD], and associated eigenvalues, were generated using DTIfit that is part of FSL's diffusion toolkit (FDT). FA maps for each subject were also visually inspected for quality and for any movement artefact and discarded from further analysis if the images were not satisfactory.

Statistical Analysis

Voxelwise statistical analysis of the FA and MD data was carried out using TBSS (Tract-Based Spatial Statistics, [R4]), part of the FSL toolkit. First, FA images were created by fitting a tensor model to the raw diffusion data using the FDT tool, and then extracted from the brain using the BET tool [R5]. All subject's individual FA images were aligned to every other one to identify the most representative image to use as the target image. The target image was then affine-aligned to the 1x1x1 mm^3 Montréal Neurological Institute (MNI) 152 standard space, and all data were then aligned into a common space using the non-linear registration tool FNIRT, which uses a b-spline representation of the registration warp field [R6].

All transformed FA images were then averaged to create a mean FA image. This mean FA image which was then thinned (skeletonized) to create a mean FA skeleton. The mean FA skeleton takes only the centres of the white matter tracts and so represents the centres of all tracts common to the group. The skeleton was thresholded by FA > 0.20 to ensure that gray matter regions were excluded from the analyses and also to reduce any cross-subjects variability where the non-linear registration has not attained a good alignment.

Each subject's aligned FA data was then projected onto this skeleton by searching for the local centre of the nearest and relevant fibre tract. This resulted in an individual FA skeleton for each subject that could then be analysed in a group comparison of TS patients versus controls using the FSL tool "Randomize" with 500 permutations. The resulting data was analysed using voxelwise, cross-subject, statistics by applying a control group vs. patient group unpaired t-test. The voxelwise cross-subject statistics were also performed on the MD maps for all subjects, and comparisons of MD maps between groups were made. Significant clusters were thresholded at a significance of $p<0.05$ that was fully corrected for multiple comparisons with Threshold-Free Cluster Enhancement (TFCE).

The results of the statistical analysis were thresholded and thickened, using the script tbss_fill, to fill the image out into the local tracts for visualisation. Identification of the white matter tracts of significant differences between groups was performed using the JUH ICBM-DTI-81 White-Matter Labels atlas and the JUH White-Matter Tractography atlas in the FSL atlas tools toolbar. The white matter tractography atlas [R7] was used for verification of anatomical location.

Regions of Interest (ROIs)

Regions of interest (ROIs) were defined by the statistical comparison of MD values in TS subjects compared to controls. ROI masks were created in FSL based on the statistical maps of areas of significant difference between groups. The TS group exhibited *reduced* FA values and/or *increased* diffusivity: particularly in the cingulum, corpus callosum, anterior thalamic radiations, forceps minor, external capsule, superior longitudinal fascicle, retrolenticular part of the internal capsule, and the corticospinal tract. Individual seed regions as defined from the group analysis were back projected into each individual's native space using the parameters derived from spatial normalisation. This allowed the masked ROIs to be projected onto each individuals FA map. The masked region could be extracted from the individual subject's FA map and a mean value extracted to provide individual FA values for each ROI. Two ROIs were selected, *a priori,* the corpus callosum (CC) and the forceps minor (FM). Mean FA values, for each TS subject, for each of these ROI were correlated with that individual's tic severity score (YGTSS). The same process was used on MD maps to extract and examine correlations of MD values with clinical scores.

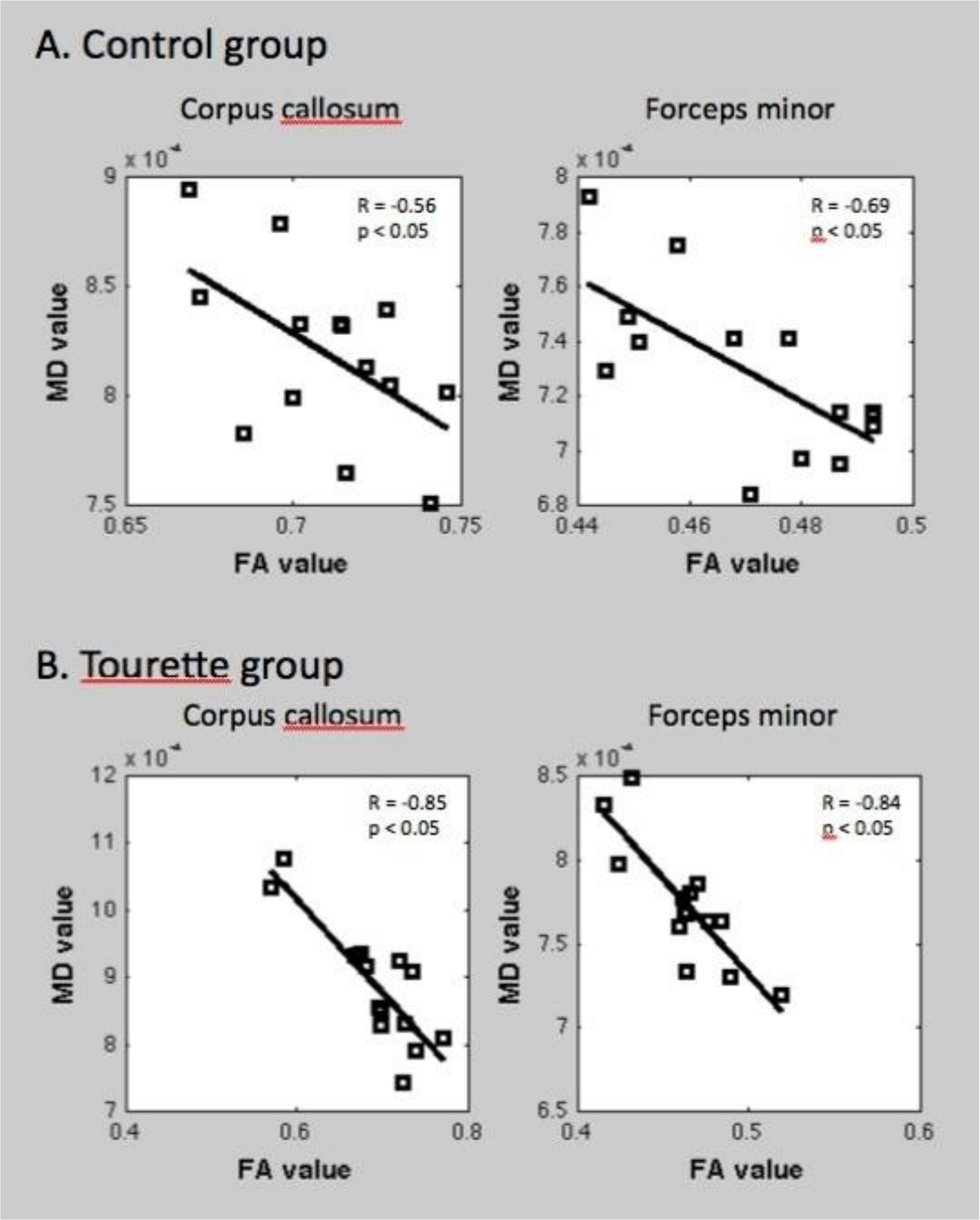

Figure S1. Scatter Plots Illustrating the Statistically Significant Negative Linear Relationship between Individual FA and MD Values

(A) The upper panel shows the linear relationship between FA and MD values for the control group [CS] in the corpus callosum and forceps minor ROIs.

(B) The lower panel shows the linear relationship between FA and MD values for the Tourette group [TS] within the corpus callosum and forceps minor ROIs.

Supplemental Results

Correlation with Clinical Scores

Correlation (Pearson) and regression analyses revealed that the WM microstructure (FA and MD) in two areas (ROIs) selected *a priori* -- the corpus callosum [CC] and the right forceps minor [FM] (a WM pathway that connects the lateral and medial areas of the prefrontal cortex) -- were strongly associated with clinical measures of tic severity (Yale motor score).

Relationship between FA and MD

FA and MD values may be inversely related: and this would make sense physiologically if the reduction in MD and increase in FA within each a particular voxel were explained by the same process (e.g. increased number of white-matter fibres passing through the voxel or increased myelination of fibres). To determine whether the observed patterns of increased MD and decreased FA (and vice versa) are observed in the *same* individuals, we examined the association between FA and MD for all participants. These data are presented in Figure S1 which demonstrates that, for both controls and TS patients, and for both CC and FM ROIs, there is a strong association between FA and MD such that individuals displaying high FA values also have low MD scores and vice versa (minimum $R = -0.56$, $p < 0.05$). Previous studies have confirmed that this inverse relationship between FA and diffusivity is independent of age and is not due to sampling artefacts (e.g., partial voluming) [R8]. It is likely that during adolescence the increase in FA and decrease in diffusivity arises from decreased intra-voxel interstitial fluid as a result of an increased number of white-matter fibres or increased myelinisation [R8]).

Effects of Age and IQ

As noted in the main text, Pearson correlation coefficients were computed and revealed that, for the TS group, there were significant correlations between the FA and MD values observed in the corpus callosum ROI and clinical measures of tic severity (Yale motor score). The correlations between the FA and MD values in the forceps minor ROI and Yale motor score were in the same direction but were only marginally statistically significant.

There were no significant correlations of FA or MD with either IQ or Age for the TS patients: however to be entirely sure that Age could not be a confounding factor, we carried out additional stepwise linear regression analyses in which Age and CC FA were entered as separate predictors of Yale motor score. These analyses confirmed that *Age alone* was not a significant predictor of Yale score ($t = 0.86$, $R^2 = 0.06$, $F = 0.73$, $p = 0.41$) but CC FA was ($t = 2.49$, $R^2 = 0.34$, $F = 6.22$, $p = 0.03$). Importantly the combination of Age and CC FA did not increase the power of the linear model but in fact reduced the amount of variance explained ($t = 2.26$, $R^2 = 0.36$, adjusted $R^2 = 0.24$, $F = 3.05$, $p = 0.09$).

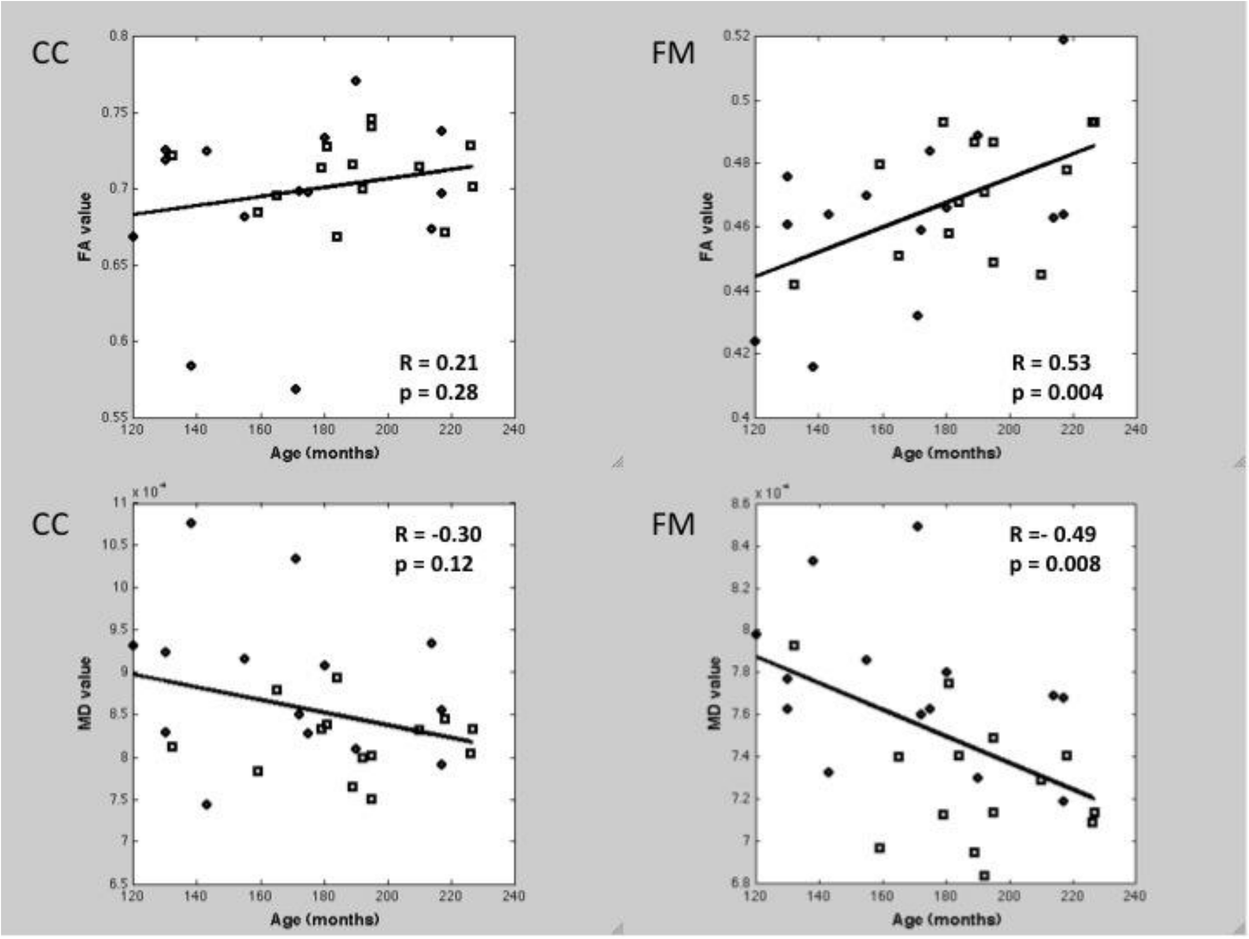

Figure S2. Scatter Plot Illustrating that There Is a Statistically Significant Linear Relationship between Age and Measures of White-Matter Microstructure within the Forceps Minor (FM) ROI (Right), but Not the Corpus Callosum (CC)

The upper panels show the linear relationship between age and FA values, and the lower panels the relationship between age and MD values. As the groups do not differ in age, linear regression analyses were performed on the whole sample. Square symbols signify members of the CS group and diamond symbols members of the TS group.

Correlation of ROI FA and MD Values with Behavior

The mean ages of the Tourette and control groups did not differ from one another. However, the FA and MD values in the FM ROI, but not the CC ROI, were significantly correlated with age (Figure S2). To investigate whether RT performance in the behavioral task-switching paradigm was predicted by the WM microstructure in the CC and FM, we felt it necessary to first control for any effect of age. We performed a series of stepwise linear regression analyses that investigated, for each group separately, and for each ROI, the degree of association between behavioral RT and WM microstructure (i.e., FA and MD values) *independently of Age*. The results of these analyses are outlined below.

CS Group: Stepwise Linear Regression Analyses

Stepwise linear regression analyses revealed that Age did not predict the RT data (i.e. Congruent-Switch RT, Congruent RT Switch costs; Incongruent-Switch RT, Incongruent RT Switch costs) for the CS group (maximum $R^2 = 0.17$, $F = 2.36$, $p > 0.1$). By contrast, RT data was significantly predicted by corpus callosum [CC] MD as follows: Congruent-Switch RT ($R^2 = 0.28$, adjusted-$R^2 = 0.22$, $F = 4.68$, $p < 0.05$); Congruent RT Switch costs ($R^2 = 0.39$, adjusted-$R^2 = 0.33$, $F = 7.51$, $p < 0.02$); Incongruent RT Switch costs ($R^2 = 0.28$, adjusted-$R^2 = 0.22$, $F = 4.57$, $p < 0.05$).

Importantly, the predictive power of CC MD was substantially reduced if Age was also entered into the stepwise linear regression. The linear regression analyses also confirmed that WM values from the forceps minor [FM] did not predict behavioral RT scores for the CS group.

TS Group: Stepwise Linear Regression Analyses

Stepwise linear regression analyses revealed that, for the TS group, Age *was* a significant predictor of behavioral RT scores as follows: Congruent-Switch RT ($R^2 = 0.38$, adjusted-$R^2 = 0.33$, $F = 7.29$, $p < 0.02$); Incongruent-Switch RT ($R^2 = 0.37$, adjusted-$R^2 = 0.32$, $F = 7.01$, $p < 0.02$).

Behavioral RT scores were also significantly predicted in the TS group by FA and MD values in the forceps minor [FM] but *not* the corpus callosum (Congruent-Switch RT x FM FA value: $R^2 = 0.33$, adjusted-$R^2 = 0.28$, $F = 5.95$, $p < 0.03$; Incongruent-Switch RT x FM FA value: $R^2 = 0.41$, adjusted-$R^2 = 0.36$, $F = 8.18$, $p < 0.01$; Incongruent-Switch RT x FM MD value: $R^2 = 0.29$, adjusted-$R^2 = 0.22$, $F = 4.63$, $p < 0.05$).

Importantly, the variance in behavioral RT scores accounted for by FM WM microstructure was shown to be *independent of Age*. This was demonstrated by the fact that FM WM microstructure remained a significant predictor of behavioral RT when entered into a stepwise linear regression *after* Age had been entered (i.e. Congruent-Switch RT x FM FA value: $R^2 = 0.46$, adjusted-$R^2 = 0.37$, $F = 4.74$, $p < 0.03$; Incongruent-Switch RT x FM FA value: $R^2 = 0.51$, adjusted-$R^2 = 0.42$, $F = 5.6$, $p < 0.02$).

To summarize: the above findings demonstrate that, *for the CS group only*, WM microstructure within the corpus callosum significantly predicts all behavioral RT measures. However, this is not the case for the TS group and it is also *not* the case for the WM microstructure of the forceps minor. By contrast *for the TS group only*, WM microstructure in the forceps minor, but not the corpus callosum, significantly predicts behavioral RT.

Study 3: Functional Magnetic Resonance Imaging Study

Supplemental Experimental Procedures

Participants

Ten young patients with Tourette syndrome participated in the study (8 male, 2 female, age 13 years 9 months [± 1 year 9 months]). The control group comprised fifteen age-matched neurologically normal individuals (age 14 years 4 months [± 1 year 11 months]). Patients were recruited through the Tourette syndrome clinic in the Child and Adolescent Psychiatry Department at Queens Medical Centre, Nottingham. Informed consent was obtained from each participant before the start of the experiment. Participants who had a clinical diagnosis of ADHD or OCD were excluded from the sample. Current tic severity for the TS patients was assessed on the day of testing using the Yale Global Tics Severity Scale [**R1**]. Ethical approval for the study was obtained from the Nottingham Healthcare Trust, and informed written consent was provided by each individual prior to participation.

Task Paradigm

The task used was conceptually identical to that presented in Experiment 1, but the timings between events, and the total number of trials presented, were adapted for use within a fMRI paradigm as outlined below.

Stimuli were presented using Matlab (R2006a, version 7.2.0.232) installed on a Windows-based laptop and back-projected onto a screen which the participants were able to view from within the MR scanner using a mirror mounted above the participant's head and fixed to the head coil. Participants were required to press a button on a MRI-compatible button box with either their left or right thumbs.

A white fixation cross was presented for approximately 1000ms before the onset of each trial. After approximately 1000ms, an arrow was displayed in the centre of screen. If the arrow was green, the participant was instructed to press the button on the side that corresponded to the direction in which the arrow pointed (Congruent trials). By contrast, if the arrow was red then the participant was instructed to press the button that was on the opposite side to the direction in which the arrow was pointing (Incongruent trials). The arrow stimuli disappeared from the screen as soon as the participant responded with a button press and the screen remained blank for approximately 7000ms before the following trial commenced. The order for Congruent and Incongruent trials was randomized.

MR Imaging Parameters

All structural and functional MRI data were acquired on a 3T Philips Achieva Scanner (Best, Netherlands) using an 8-channel SENSE head coil. The high resolution T1 weighted structural images were acquired using a magnetization prepared gradient echo sequence (MPRAGE, 256 slices, FOV=256mm, 160 transversal slices) with a resolution of 1mm isotropic. The functional images were acquired using echo-planar imaging (EPI) sensitive to BOLD contrast. T2* weighted BOLD images were acquired using the following parameters: FOV 256mm; slice acquisition voxel size = 3x3x3 mm; 36 slices; matrix size = 96 x 96; flip angle = 80; TR = 2200ms; and TE = 40 ms. The slices were contiguous and

taken in a descending order. The experimental runs of the saccadic task consisted of 225 volumes and the runs for the manual task of 300 volumes.

Image Preprocessing

Brain Voyager QX 1.10.2 software (Brain Innovation, Maastricht, The Netherlands) was used for fMRI data analysis. Preprocessing of the fMRI datasets consisted of: (1) 3D head motion correction; (2) slice scan timing correction to correct for the temporal difference in acquisition of different slices; (3) spatial smoothing (Gaussian kernel of FWHM 4mm); and (4) linear trend and high-frequency component removal (up to and including 3 cycles in the time course). Whereas ten TS participants were recruited for this study, data from two TS participants had to be excluded from further analysis due to excessive head motion.

Behavioral Analyses

Analyses of the RT responses confirmed that RTs were substantially slower for Incongruent trials compared to congruent trials (means: Incongruent = 622 [±27]ms, Congruent = 594 [±25]ms; $t = 3.21$, $p < 0.005$) and slower for task Switch trials compared to task Repeat trials (means: Switch = 629 [±30]ms, Repeat = 586 [±22]ms; $t = 3.78$, $p < 0.001$). *A priori*, between-group, contrasts revealed that there were no statistically significant differences in RT between the groups for any of the experimental conditions (maximum $t < 1$, $p > 0.1$).

fMRI and Anatomical Data Analysis

Anatomical images were transformed into the Talairach coordinate system and co-registered with each fMRI dataset. Regional activation maps were obtained using a single-subject GLM (General Linear Model) for each individual. For each task, four predictors were defined: Incongruent-switch (IS), Incongruent-repeat (IR), Congruent-switch (CS) and Congruent-repeat (PR). Initial second level analyses involved calculating three-dimensional statistical parametric maps with separate-subject predictors for the group, using a fixed effects GLM analysis (FFX). A FFX analysis was conducted due of the relative small number of individuals participating in the study, but more importantly because the theoretical premise being tested (and already partly confirmed by the DWI data reported in Study 2) is that when performing the behavioral task, *different* brain areas will become activated by the TS group compared to the CS group.

The resulting fMRI activity maps were thresholded at a t-value corresponding to $p < 0.05$ (corrected for a false discovery rate in which no more than 5% of the active voxels could be false positives [R9]) with a minimum cluster threshold of at least 20 voxels. These images were then smoothed with a 3mm Guassian kernal. Subsequent analyses involved the identification of functional regions-of-interest (ROIs), specified *a priori* from the results of the DWI study (Study 2 above) in (A) the hand area of the left and right hemisphere motor cortex, and (B) bilateral regions of the prefrontal cortex immediately adjacent to the forceps minor WM tract.

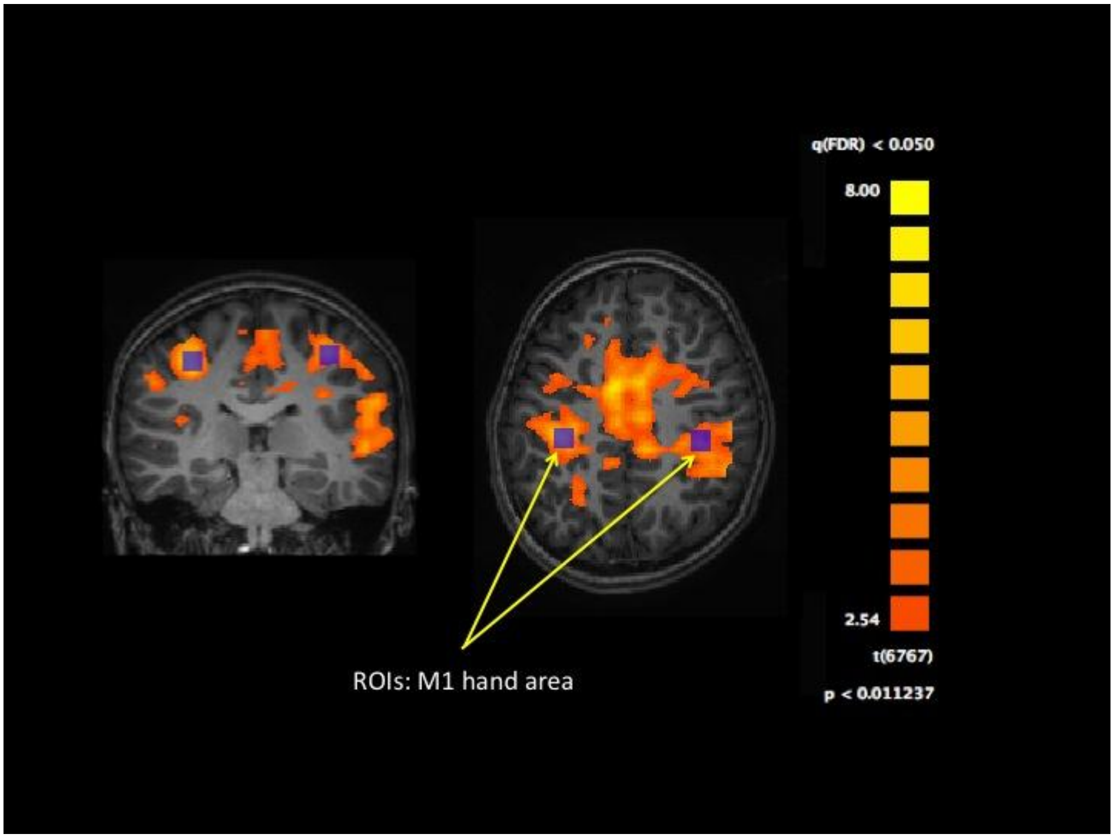

Figure S3.
Illustrates the location of the bilateral ROIs corresponding to the hand area of motor cortex (purple squares) superimposed over the fMRI BOLD activation in response to the *All conditions* > *Rest* contrast.

The location of the hand area of primary motor cortex was first identified within each hemisphere using anatomical landmarks. A 10mm^3 ROI was then centred within each hemisphere around the location that corresponded to the peak voxel activated for the *All Conditions* > *Rest* contrast (Figure S3). The location of the bifurcation of the forceps minor WM tract within the prefrontal cortex was first identified within each hemisphere using anatomical landmarks. A 10mm^3 ROI was then centred within each hemisphere around the location that corresponded to the peak voxel activated for the *Incongruent* > *Congruent* contrast.

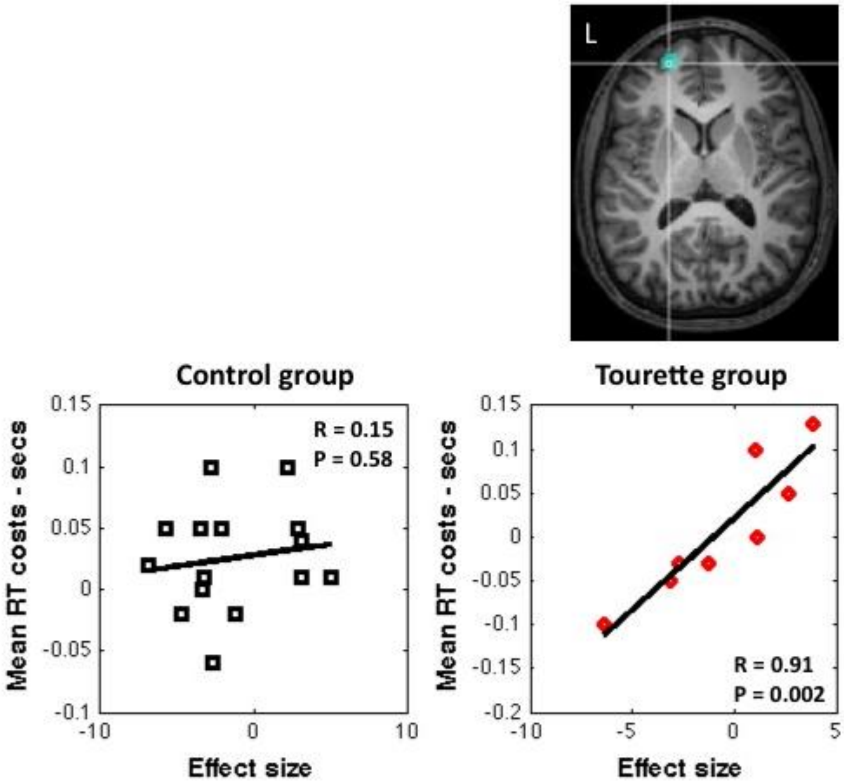

Figure S4.

Scatter plot illustrating, for each group, the linear relationship between fMRI BOLD responses within the left prefrontal cortex ROI and individual RT cost values for the Incongruent condition of the manual task-switching paradigm.

Supplemental Results

Correlation between Left Prefrontal BOLD and Behavioral RT Costs

To investigate the relationship between the fMRI BOLD response in the left prefrontal cortex ROI and behavior in the manual task-switching paradigm, we examined the linear relationship, for each group, between individual RT cost values (Switch minus Repeat trials) for the Incongruent condition, and individual parameter estimates for the appropriate fMRI BOLD contrast (i.e., Incongruent Switch trials > Incongruent Repeat trials). These data are presented in Figure S4. Inspection of this figure clearly shows that for the *TS group only*, the fMRI BOLD response is very strongly, and positively, linearly associated with RT costs (R = 0.91, p = 0.002). Thus, increased BOLD activation in the left prefrontal ROI is also associated with high conflict situations and, in the TS group, with increased RT costs. In contrast, the linear relationship between the BOLD response in the right prefrontal cortex ROI and RT switch costs is largely absent for the control group and is not statistically significant.

Supplemental References

R1. Leckman JF, Riddle MA, Hardin MT, Ort SI, Swartz KL, Stevenson J, Cohen DJ. (1989). The Yale Global Tic Severity Scale: initial testing of a clinician-rated scale of tic severity. J Am Acad Child Adolesc Psychiatry 28: 566-73.

R2. Mueller SC et al. (2006) Enhanced Cognitive Control in Young People with Tourettes Syndrome. *Current Biology* 16, 570-573.

R3. Jackson GM, Mueller SC, Hambleton K, Hollis CP. (2007). Enhanced cognitive control in Tourette Syndrome during task uncertainty. Experimental Brain Research 182:357-64.

R4. Smith SM, Jenkinson M, Johansen-Berg H, Rueckert D, Nichols TE, Mackay CE, Watkins KE, Ciccarelli O, Cader MZ, Matthews PM, Behrens TE. (2006). Tract-based spatial statistics: voxelwise analysis of multi-subject diffusion data. Neuroimage. 31:1487-505.

R5. Smith SM. (2002). Fast robust automated brain extraction. Human Brain Mapping 17: 143-55.

R6. Rueckert D, Sonoda LI, Hayes C, Hill DL, Leach MO, Hawkes DJ. (1999). Nonrigid registration using free-form deformations: application to breast MR images. IEEE Trans Med Imaging 18: 712-21.

R7. Wakana S, Jiang H, Nagae-Poetscher LM, van Zijl PC, Mori S. (2004). Fiber tract based atlas of human white matter anatomy. Radiology 230:77-87.

R8. Pfefferbaum A, Sullivan EV. (2003). Increased brain white matter diffusivity in normal adult aging: relationship to anisotropy and partial voluming. Magn. Reson. Med. 49: 953-61.

R9. Storey JD, Tibshirani R. (2003). Statistical significance for genome wide studies. Proc. Natl. Acad. Sci. U S A. 100: 9440-9445.
